# Measuring quality of facility-based ITN distribution in Ghana

**DOI:** 10.1186/s12936-023-04626-y

**Published:** 2023-08-02

**Authors:** Luigi Nuñez, Malia Skjefte, Obed E. Asamoah, Prince Owusu, Keziah L. Malm, Jane E. Miller

**Affiliations:** 1 PMI VectorLink, PSI, Washington DC, USA; 2 PMI VectorLink, PSI, Accra, Ghana; 3 National Malaria Elimination Programme, Accra, Ghana

**Keywords:** Malaria, ITN, ANC, CWC, Supervision, Routine distribution, Continuous distribution, Pregnant women, Children, Ghana

## Abstract

**Background:**

Continuous distribution channels are effective methods to deliver malaria interventions such as insecticide treated nets (ITNs) to pregnant women attending antenatal care clinics and children under five attending immunization visits. Facility-based and provider-based checklists were used during supportive supervision visits to measure the quality of facility-based services and interventions. This study looks at ITN distributions at health facilities in Ghana, with the aim of providing insights on how quality can be measured and monitored.

**Methods:**

Various quality improvement approaches for malaria services occur in Ghana. Selected indicators were analysed to highlight the similarities and differences of how the approaches measured how well the channel was doing. Generally, the approaches assessed (1) service data management, (2) logistics data management, and (3) observation of service provision (ITN issuance, malaria education, ITN use and care education). Two approaches used a binary (Yes/No) scale, and one used a Likert scale.

**Results:**

Results showed that most data reported to the national HMIS is accurate. Logistics data management remained an issue at health facilities, as results showed scores below average across facility stores, antenatal care, and immunization. Though the supervision approaches differed, overall results indicated that almost all eligible clients received ITNs, data were recorded accurately and reported on-time, and logistics was the largest challenge to optimal distribution through health facilities.

**Conclusion:**

The supervision approaches provided valuable insights into the quality of facility-based ITN distribution. Ghana should continue to implement supportive supervision in their malaria agenda, with additional steps needed to improve reporting of collected data and increase the number of facilities visited for supportive supervision and the frequency. There were various supervision approaches used with no clear guidance on how to measure quality of facility-based ITN distribution, so there is also need for the global community to agree on standardized indicators and approaches to measuring quality of facility-based ITN distribution. Additionally, future studies can review the effect of multiple rounds of supervision visits on the quality of ITN distribution as well as understand the facilitators and barriers to scaling up supervision of facility-based ITN distribution.

## Background

Global efforts to reduce the burden of malaria include the large-scale distribution of insecticide-treated nets (ITNs) through mass campaigns and continuous distribution channels. These channels include distribution via routine health services where nets are given to pregnant women in antenatal care (ANC) clinics and to children under 5 years of age during visits to Expanded Programme of Immunization (EPI) clinics. According to the World Health Organization (WHO) 2022 World Malaria Report, 35 countries in the WHO African Region distributed ITNs through ANC in 2021, while 30 countries distributed ITNs through EPI clinics in the same year [1]. A review by Theiss-Nyland et al. [2] found that, across 25 sub-Saharan African countries, “an average of 54% of children slept under an ITN” with both ANC and EPI distribution, compared with 34% with ANC only and 24% with no facility-based distribution. While analysing Demographic and Health Survey (DHS) data across multiple countries, the authors also calculated “a 13% increase in net use among children under five, on average, with each additional ITN distribution policy” [2]. Thus, having these channels operating efficiently will improve the positive contribution of these channels to net access and use.

By 2025, Ghana aims to protect at least 80% of its population at risk of malaria infection through the use of effective malaria prevention interventions, including vector control methods such as ITNs [3]. Continuous distribution of ITNs through health facilities serves as a primary channel for maintaining ITN access that is achieved through triennial ITN mass distributions and ensuring continuous access to ITNs for pregnant women and children under one year of age. In Ghana, continuous distribution of ITNs through health facilities has been implemented in all districts since 2012 through distribution of ITNs to pregnant women receiving ANC and children receiving the Measles-Rubella Vaccine 2 (MR2) at EPI services (through child welfare clinics [CWCs]) [4]. There have also been four rounds of mass ITN distribution campaigns since 2010. School-based distribution, another continuous distribution channel alongside facility-based distribution, is conducted annually. Additionally, health workers have been trained to provide education on ITN use and care, which is key to consistent use and care of the ITNs, such as the use of media messaging in malaria prevention [5].

Quality improvement of health service delivery, as defined by the WHO, is an “approach to the improvement of service systems and processes through the routine use of health and program data to meet patient and programme needs” [6]. The literature defines quality improvement, similar to the WHO definition, as a systematic approach to improve healthcare services and, consequently, patient health outcomes by strengthening knowledge, skills, and infrastructure [7–12]. Supportive supervision is one approach to quality improvement, commonly described as a process to help staff improve their ability to offer high-quality healthcare services through a respective, open, communicative, collaborative, and problem-solving lens and typically includes using data to monitor progress towards goals [13–17].

Supportive supervision is considered an effective method for identifying barriers to optimal service delivery and improving malaria case management in sub-Saharan Africa. For example, in a 2018 study in Uganda, authors noted that a high score in health facility readiness was associated with a reduced risk of severe malaria outcomes and that the biggest barrier to achieving high facility readiness was stock shortages of basic amenities and essential medicines [18]. Another study in Mozambique found discrepancies between the data collected in the facilities’ registry and the data reported into the District Health Information Management System (DHIMS) [19]. A 2013 study by Bello et al. [20] showed the effectiveness of supportive supervision in improving the delivery of primary care services in Jos, Nigeria. Another study by Martin et al. [21] predicted that clinical performance would improve by 6.3% after three supportive supervision visits during which healthcare workers received education and on-site mentoring. Additional studies have also analysed the effect of supportive supervision on the adherence to the WHO malaria case management guidelines, including malaria microscopy [22, 23].

However, literature on the topic of quality improvement of ITN distribution services at health facilities does not exist. Unlike other malaria services, which have step-by-step procedures endorsed at the global level, global guidelines at the health facility for optimal ITN distribution are lacking. Although the only guide for implementing facility-based ITN distributions recommends supervision to be led by the Ministry of Health (MOH) and use a checklist to “help supervisors to approach the supervision visit systematically and to review point by point each of the areas under review,” it does not advise on what should be assessed during the supervision visit for assessing the quality of facility-based ITN distribution [24]. This gap makes it difficult to consistently measure the quality of facility-based ITN distribution and propose indicators that could measure performance.

The U.S. President’s Malaria Initiative (PMI) VectorLink Project supported the National Malaria Elimination Programme (NMEP) to develop a standardized supportive supervision checklist for measuring the quality of facility-based ITN distribution based on one supervision checklist piloted by VectorLink in Ghana. The checklist collects data on service data management, logistics data management, and observation of ITN issuing to clients. The checklist is flexible in that it can be incorporated into existing supportive supervision checklists. For example, the section on service data management can be integrated into other data quality checklists, and observation of ITN issuance can be incorporated into existing ANC or CWC supervision checklists. The project also proposed indicators that can be monitored using the data generated by this checklist. The checklist has been used in Cameroon, Ghana, and Niger.

Ghana’s version of the Ministry of Health called the Ghana Health Service (GHS) monitors facility-based ITN distribution using analyses of key performance indicators which are reported through monthly facility reports and indicators assessing quality of distribution through supervision. Through two supportive supervision approaches (Outreach, Training, and Supportive Supervision [OTSS] which focuses only on malaria-related services and Integrated Supportive Supervision [ISS], which includes multiple disease areas such as malaria, HIV, and family planning) and analysis of routine District Health Information Management System 2 (DHIMS2) data, district, regional, and national teams identify successes and challenges in implementation and generate feedback to health facility staff for continuous improvement.

**Fig. 1:**
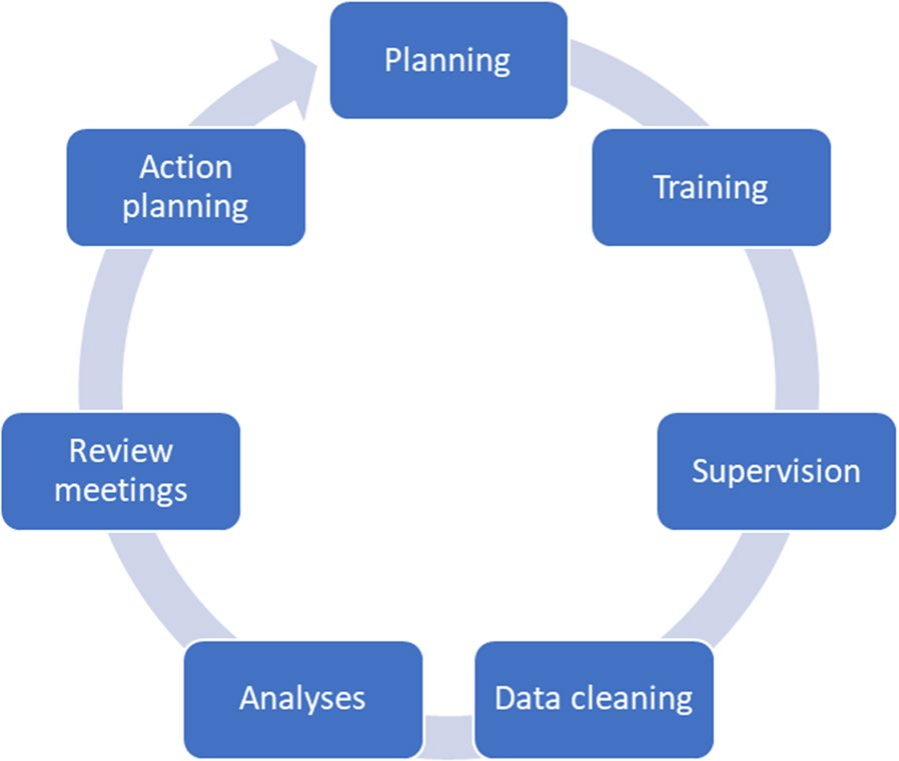
Process to implementing supervision approaches

Both approaches followed a pre-established series of activities for every round of supportive supervision. Planning was necessary to ensure an adequate budget and timelines for the successful implementation of each supervision approach. Then, training took place to ensure actors at each level have the required skills to implement the approach, including use of digital tools to complete the competency-based checklists and supportive supervision skills. After training, visits to health facilities to conduct supervision took place. The use of digital tools helped to minimize data quality issues, though checks of the data are required to identify duplicates for removal and expedited analyses through automated dashboards. Additional analyses, such as presentations and reports, were done. Meetings were then held to review identified areas of strength and areas for improvement. The meetings led to discussions and agreed-upon action items. This series of activities is outlined in Fig. 1 and repeated as needed based on available resources.

OTSS is a quarterly supportive supervision approach in which supervisors of health facilities use facility-based and provider-based checklists to measure the quality of malaria-related services and interventions. It includes supporting facility staff within their working setup, providing feedback, and strengthening documentation of services. The aim of OTSS is to enhance quality improvement in malaria prevention and case management. Observation, on-the-job training, feedback, action plans, and logistical support are activities performed during OTSS. The NMEP uses this approach to build the capacity of health care providers in malaria prevention and control. An OTSS team generally includes a clinician, public health nurse/midwife, biomedical scientist/lab professional, malaria focal person, and health information officer. The number of officers in a team usually depends on the type/level of the health facility. The modules for OTSS in Ghana include topics of clinical observation, adherence to case management protocols, pharmacy assessment, general OTSS implementation, microscopy assessment, rapid diagnostic test (RDT) observation, intermittent preventative treatment in pregnancy (IPTp) implementation, data management, and routine ITN distribution.

Also implemented quarterly, ISS involves visits to health facilities by trained teams of supervisors who conduct monitoring of all clinical services including malaria management and reinforce they are to be carried out in accordance with national guidelines to achieve expected results. Supervisors also provide on-the-job coaching and recommendations for improvement. The GHS Institutional Care Division led this supportive supervision approach until 2021, when component. For ISS, the country converted all paper-based supportive supervision tools to an Android DHIS2-based app called the Health Network Quality Improvement System (HNQIS). The ISS checklist has modules including a COVID-19 assessment, management systems in health facilities service delivery sites, public health, clinical care, reproductive and child health, and monitoring and evaluation (M&E). The ITN component of the ISS checklist assesses distribution during ANC services and is a sub-section of the Reproductive and Child Health and Nursery and Midwifery Module. It also assesses distribution at CWC, which is a sub section of the Public Health Module. The ITN ISS checklist assesses quality of distribution, stock management and issuance of ITNs to eligible clients at both ANCs and CWCs, and education on use of and care for ITNs at CWCs.

This paper provides an overview of the indicators used to measure the quality of ITN distribution at health facilities in Ghana. First, the indicators used to measure quality of facility distribution of ITNs via the two supervisory approaches are listed. Then, examples of how these indicators can be used are shown, and strengths and weaknesses of these indicators and the supervisory approaches are discussed. Finally, the paper concludes with a call to action for the global community, especially MOHs and national malaria programmes, to come to a consensus about how to appropriately measure quality of ITN distribution at health facilities.

## Methods

Among the larger OTSS team, the Routine ITN distribution module of OTSS was usually conducted by malaria focal person. It consisted of three core components which assessed the distribution of nets at ANC and CWC. The first component, service data management, included data from registers, monthly reporting forms, and DHIMS2 for the three months before the supportive supervision visits to ANC and CWC. The second component, logistics data management, included the review of logistics and inventory control of ITN at health facilities (stores, ANC, and CWC) and district health management stores. The checklist helped to ascertain whether facilities were keeping to the minimum threshold of ITNs to avoid stock-outs and regularly updating bin cards and other inventory control forms. Supervisors were expected to coach health workers on any areas of inconsistency. The last component, observation, assessed ITN issuance and education provided on ITN use and care, as well as malaria education. Supervisors were trained to coach health workers on best practices for ITN issuance and provision of education on ITN use and care if the health worker performed poorly. Assessments were entered into an Android, DHIS2-based app called the Electronic Data System (EDS) for reporting and analysis. In addition, narrative reports on observations from the field were compiled by district and regional teams and submitted to the regional level for collation. Indicators reported using data from OTSS can be found in Table 1.

**Table 1:** Indicators to measure quality of facility-based ITN distribution via OTSS

Indicator	Disaggregated for data element	Disaggregated for service delivery point
Service data management		
Average overall score for service data management		√
Percentage of facility-months where register = DHIMS2	√	
Percentage of facility-months where register > DHIMS2	√	
Percentage of facility-months where register < DHIMS2	√	
Percentage of facility-months where register or DHIMS2 data missing	√	
Logistics data management		
Average overall score for logistics data management		√
Percentage of facilities with ITN inventory control card available		√
Percentage of facilities with ITN inventory control card up to date (among available)		√
Percentage of facilities with ITN inventory control card matching physical stock quantity		√
Percentage of facilities knowing their minimum stock level for the month		√
Percentage of facilities at and above minimum stock level		√
Observation of ITN issuing		
Average overall score for observation of ITN issuing		√
Percentage of observed eligible patients who received an ITN		√
Percentage of observed issued ITNs documented in patient card		√
Percentage of observed issued ITNs documented in register		√
Percentage of observed patients given education on malaria		√
Percentage of observed patients given education of ITN use and care		√
Percentage of observed patients given demonstration on hanging an ITN		√

For ISS, the ITN component of the ISS checklist assessed distribution at ANC and is a sub-section of the Reproductive and Child Health and Nursery and Midwifery Module. It also assessed distribution at CWC, which was a sub-section of the Public Health Module. The ITN ISS checklist assessed quality of distribution, stock management, and issuance of ITNs to eligible clients at both ANCs and CWCs, and education on use of and care for ITNs at CWCs. Early versions of ISS scored questions using a Likert scale of 0–3, and the 2022 round of ISS used a binary Yes/No scoring approach to increase objectivity of how the questions were answered and interpreted. Although global guidance is to use the term “ITN” instead of “LLIN” (Long Lasting Insecticide-treated Nets), many countries still use “LLINs”, and since both versions of ISS reference “LLINs”, the text was retained as per the supervision checklists. Indicators reported using data from ISS can be found in Table 2.

**Table 2:** Indicators to measure quality of facility ITN distribution via ISS

Indicator	Disaggregated for service delivery point
ANC and CWC	
Percentage of facilities which issued ITNs to 100% of eligible patients	√
Percentage of facilities whose inventory control card for ITNs matched the physical count of stock during the visit	√
Percentage of facilities with updated inventory control cards for ITNs	√
ANC specific	
Percentage of facilities with inventory control cards available for tracking ITN supply, stock levels, and consumptions at ANC	
CWC specific	
Percentage of facilities which educated parents and guardians of children receiving measles/rubella booster vaccine on ITN care and use	

OTSS indicators from February to June 2022 were analysed from 14 regions (217 districts). One round of OTSS was implemented from February to June 2022 in 14 of the 16 regions. The Bono and Eastern regions had not completed their OTSS during the analysed period. Analyses for the ISS indicators used data generated during the supervision visits taking place from January 2022 to March 2022. As of end of May 2022, all 260 districts across Ghana’s 16 regions had completed ISS with data available for 1536 facilities at ANC and for 1642 facilities at CWC. All analyses were conducted using Microsoft Excel version 16.64.

Each section outputted a score, which took the number of questions passed divided by the number of questions answered. To assess the performance of service data management, the number of facility-months (i.e., the number of facilities *x* the number of months assessed) for which data from the register was less than, equal to, or greater than the data reported in DHIMS2 was calculated, as well as the number of facility-months missing data from at least one source.

## Results

### OTSS

**Fig. 2:**
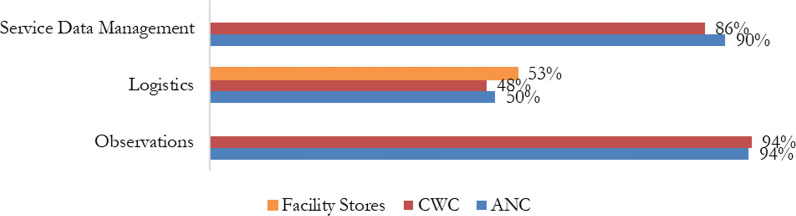
OTSS ITN Overall Scores

Analysis of the OTSS data revealed that the observation of ITN issuing and education of clients was the highest-scored component with average score of 94% at both ANC and CWC (Fig. 2). Logistics data management was lowest, with facility average scores of 48% at CWC, 50% at ANC, and 53% at facility stores. Service data management had average scores of 86% at CWC and 90% at ANC.

**Fig. 3:**
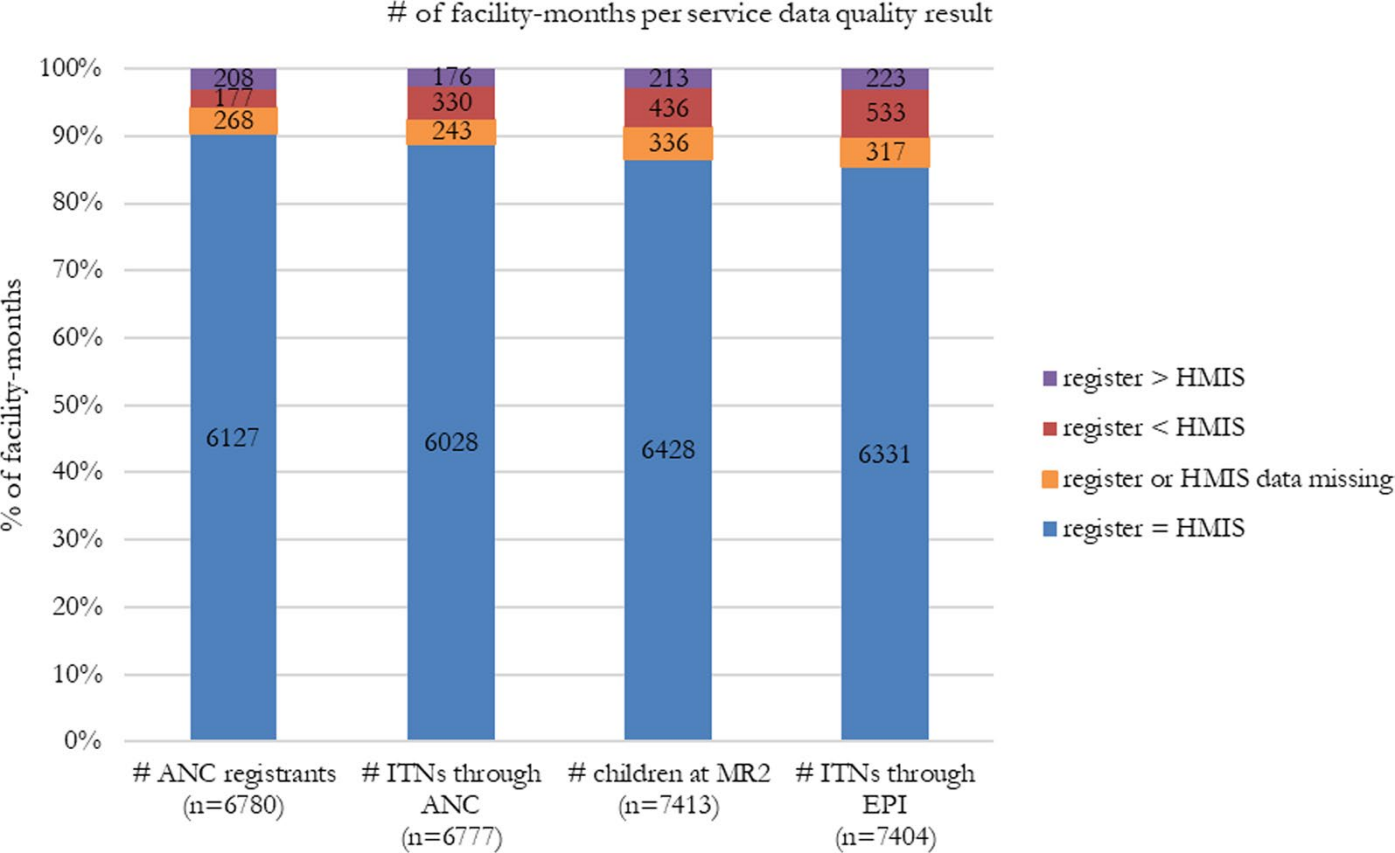
OTSS ITN service data management indicators

Supervisors were expected to assess three months of data per facility. For all four indicators captured in the register (number of ANC registrants, number of ITNs issued through ANC, number of children at MR2 visits, and number of ITNs issued through CWC), the majority of data points matched between the register and DHIMS (Fig. 3). Largely, data reported at ANCs were more accurate than at CWCs.

**Fig. 4:**
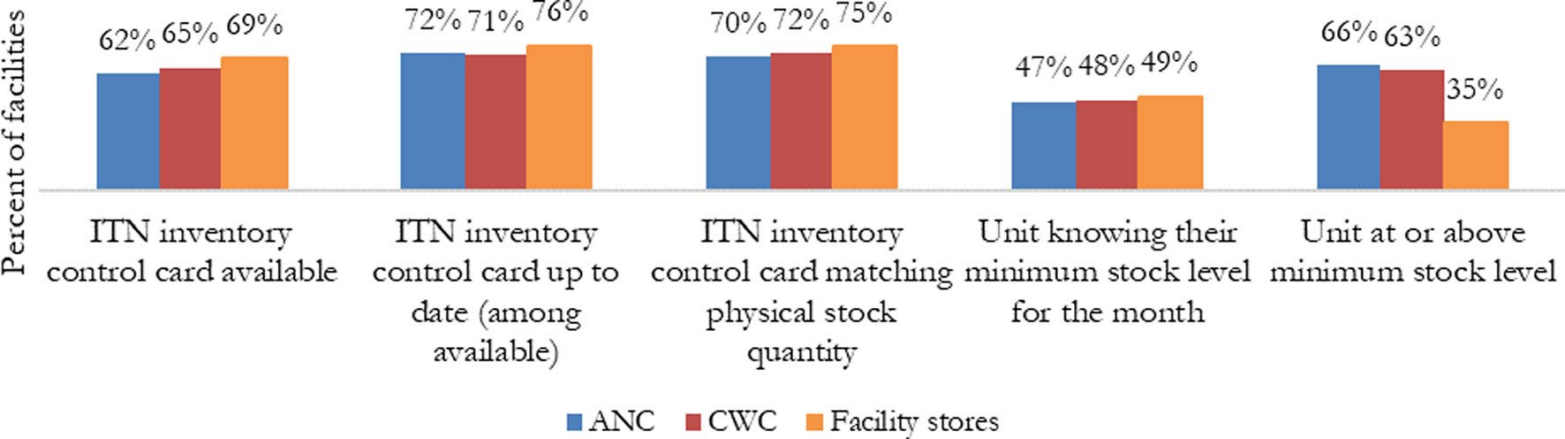
OTSS ITN logistics data management indicators

For assessing performance of logistics data management, facilities performed poorly at all three units (ANC, CWC, and facility stores). Facility stores tended to manage logistics data slightly better than at ANC and CWC, although fewer facility stores were at or above the minimum stock level during the visit (Fig. 4). Additionally, less health facilities had ITN inventory control cards available at ANC (62%) than at CWC (65%) and facility stores (69%). When the ITN inventory control card was available, similar proportions of health facilities had it up-to-date at ANC (72%) and CWC (71%). ITN inventory control cards matched physical count in 70% of ANCs, 73% of CWCs, and 75% of facility stores visited. Less than half of all ANCs, CWCs, and facility store units at visited health facilities knew their minimum stock level for the month. Finally, about two-thirds of ANC and CWC units at visited health facilities had physical stock at or above the minimum stock level.

**Fig. 5:**
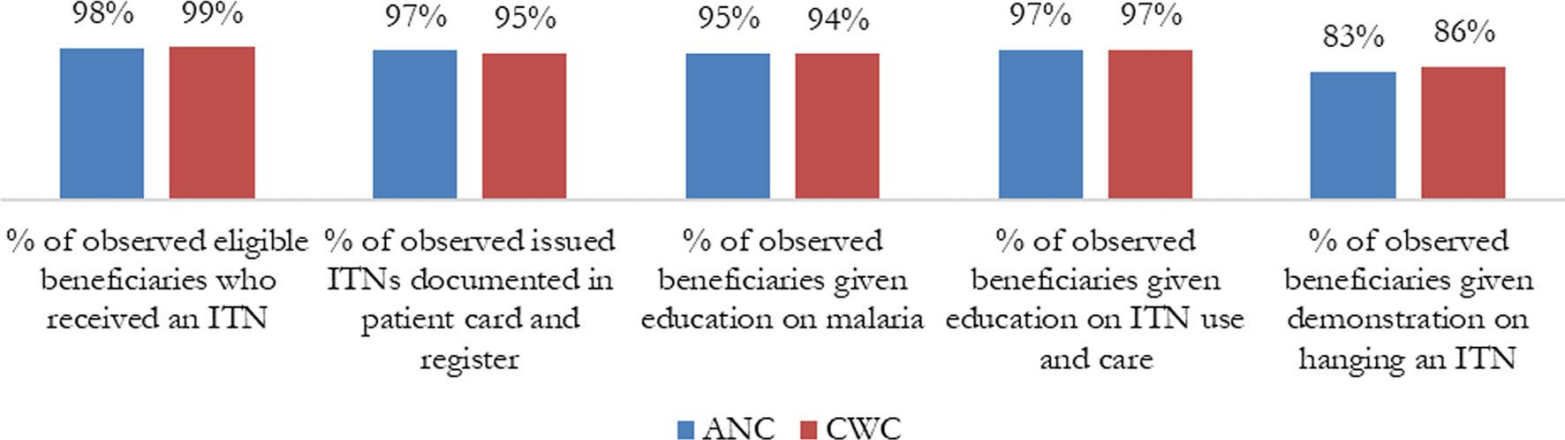
OTSS ITN observation indicators

Regarding performance of the health worker’s role in ITN issuance, almost all observed eligible beneficiaries received an ITN and were given education on malaria and ITN use and care at both ANC and CWC. In addition, almost all observed issued ITNs were documented in the patient card and register. However, only 83% and 86% of observed beneficiaries were given a demonstration on hanging an ITN at ANC and CWC, respectively (Fig. 5).

### ISS results (Likert scale)

The ITN component of the ISS checklist assessing distribution at ANCs was a sub-section of the Reproductive and Child Health and Nursery and Midwifery Module. The ITN component of the ISS checklist assessed distribution at CWCs, which was a sub-section of the Public Health Module. The ITN ISS checklist assessed the quality of distribution which includes stock management, issuance of ITNs to eligible clients at both ANC and CWC, and education on use of and care for ITNs at CWC. Data were available for ITN distribution performance at ANC for 1536 facilities and at CWC for 1642 facilities across all 260 districts. Each assessment area was scored according to the scheme as explained in Table 3.

**Table 3:** Explanation of ISS Likert Scale

Score	Description
3	Outstanding: If the criterion* is met or all criteria are met (* where there is a single criterion, 3 if the criterion is met or 0 if it is not met)
2	Good: If more than half of the criteria are met
1	Average: If half of the criteria are met
0	Poor: If less than half the criteria are met

**Fig. 6:**
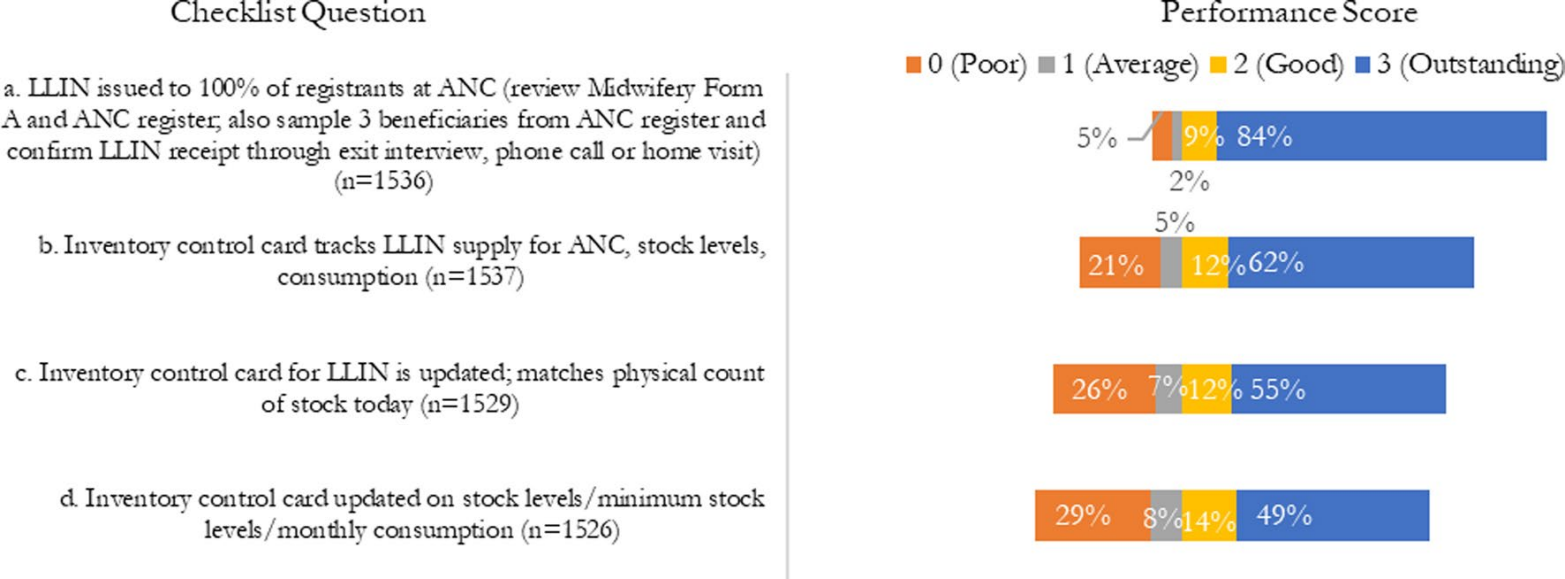
ISS Likert indicators for ITN distribution at ANC

The ISS had four questions for ITN distribution at ANC to assess performance. According to the ISS data (Fig. 6), 84% of the visited facilities received an outstanding score for “LLIN issued to 100% of registrants at ANC,” which was done via reviews of the Midwifery Form A and the ANC register as well as confirmation via phone calls, exit interviews, or home visits. Sixty-two percent of visited facilities received an outstanding score for “Inventory control card tracks LLIN supply for ANC, stock levels, consumption.” About half received an outstanding score for “Inventory control card for LLIN is updated; matches physical count of stock today” (55%) and “Inventory control card updated on stock levels/minimum stock level/monthly consumption” (49%).

**Fig. 7:**
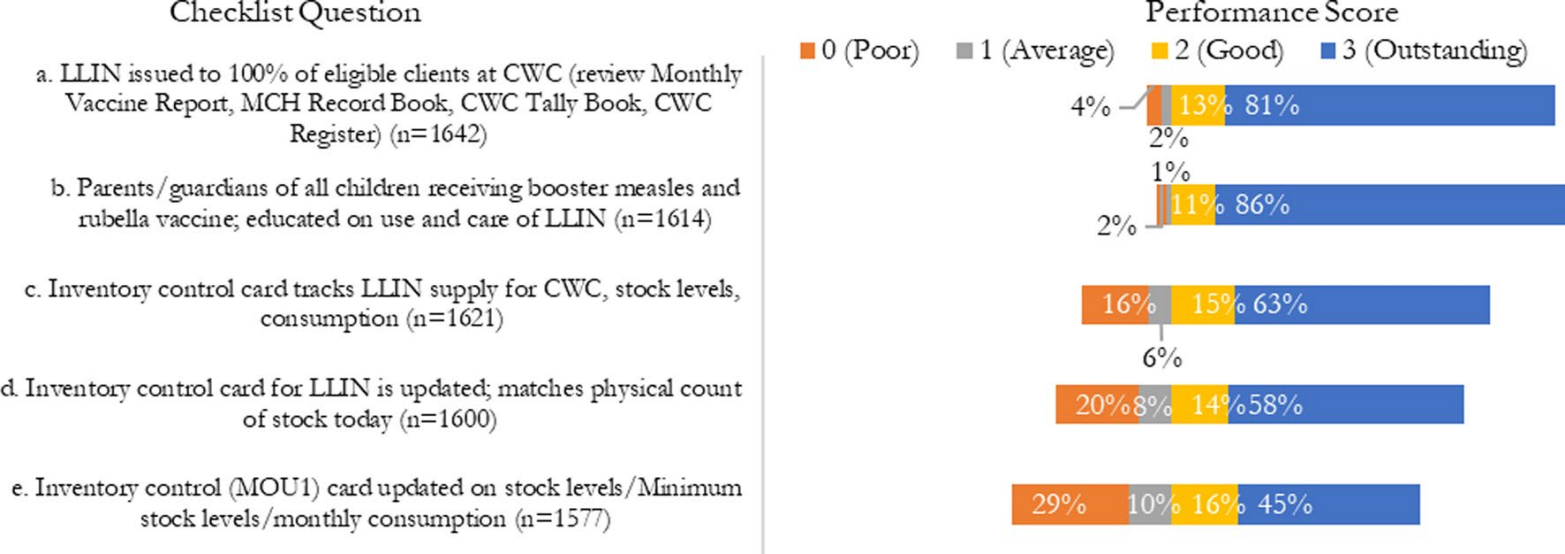
ISS Likert indicators for ITN distribution at CWC

For ITN distribution at CWC, the ISS had five questions to assess the performance. According to the ISS data from CWCs (Fig. 7), 81% of the visited facilities received an outstanding score for “LLIN issued to 100% of registrants at CWC.” The evaluation occurred by conducting reviews of the monthly vaccine reports, maternal and child health record books, CWC tally books, and CWC registers. 86% received an outstanding score for “Parents/guardians of all children receiving booster measles and rubella vaccine; educated on use and care of LLIN.” 63% received an outstanding score for “Inventory control card tracks LLIN supply for CWC, stock levels, consumption,” and 58% received an outstanding score for “Inventory control card for LLIN is updated; matches physical count of stock today.” Less than half (45%) received an outstanding score for “Inventory control (MOU1) card updated on stock levels/minimum stock level/monthly consumption.”

### ISS results (binary scale)

From January 2022, ISS was modified to use a binary scale (Yes/No) instead of a Likert scale for assessment questions to make it easier for objective interpretation. The ITN component of the ISS checklist was a sub-section of the Public Health Module, which assessed the distribution of ITNs at CWC. The ITN ISS checklist assessed the quality of distribution, stock management, and issuance of ITNs to eligible clients at both ANC and CWC, as well as education on use of and care for ITNs at CWC. ISS data was analysed to understand ITN distribution performance across ANC for 1536 facilities and CWC for 1642 facilities.

**Fig. 8:**
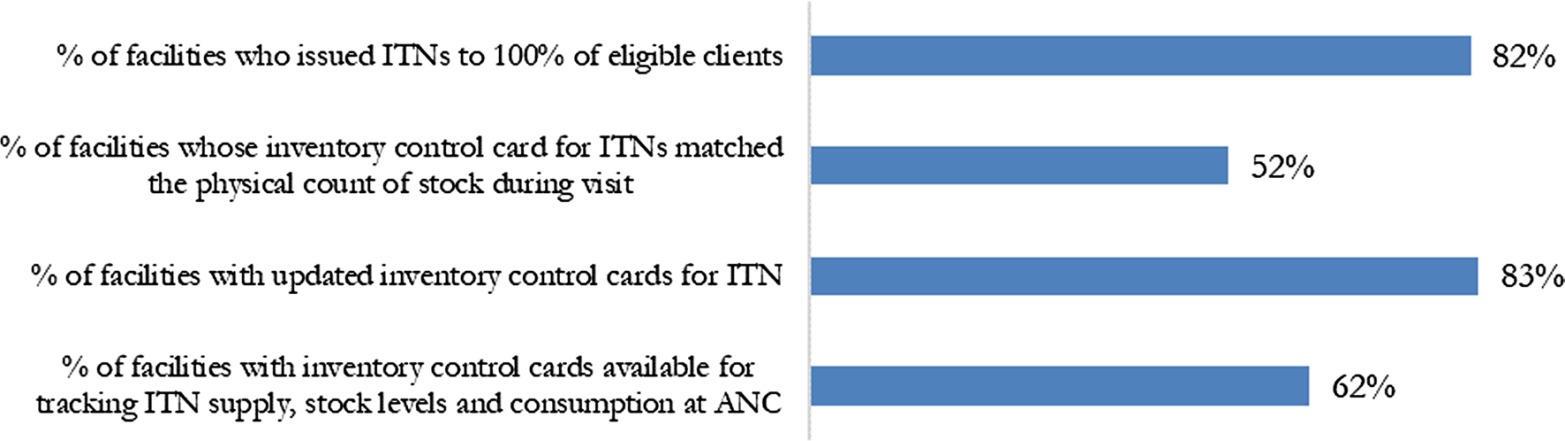
ISS Binary indicators for ITN distribution at ANC

For ITN distribution at ANC, the ISS used four questions to assess performance. When reviewing the facilities which issued ITNs to 100% of eligible clients, 82% of the assessed facilities met this performance criteria (Fig. 8). Additionally, 83% of the facilities at ANC had updated inventory control cards for ITN. The performance at ANC was lower when looking at the percentage of total facilities in which inventory cards matched the physical stock, as only 52% of facilities met this mark. Lastly, only 62% of facilities had inventory control cards available for tracking ITN supply, stock levels, and consumption at ANC.

**Fig. 9:**
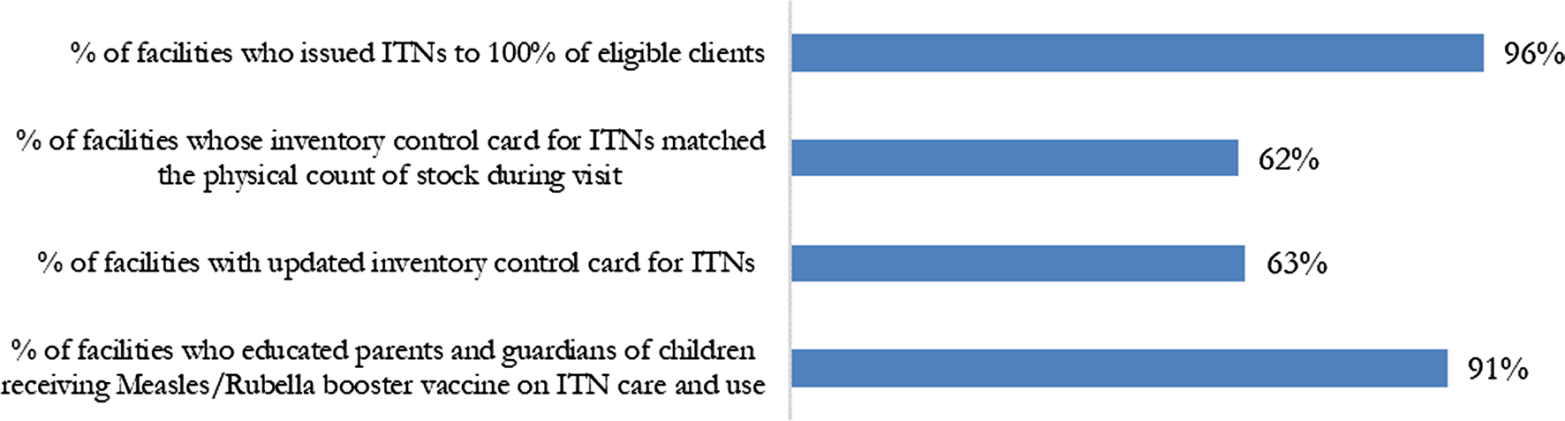
ISS Binary indicators for ITN distribution at CWC

For ITN distribution at CWC, 96% of facilities issued ITNs to 100% of their eligible clients and 91% of facilities educated parents and guardians of children receiving the MR2 booster on ITN care and use. However, only 63% of facilities had updated inventory control cards for ITNs and only 62% had inventory control cards that matched the physical stock during the visit (Fig. 9).

## Discussion

### General remarks

The three different methods of analysis demonstrated different ways to measure the quality of health facility-based ITN distribution. All three provided some data about correct adherence to issuing ITNs to eligible clients, quality of inventory management, and education given to the client. Among these three analysis methods, only OTSS provided additional data on data quality. In addition, ISS only assessed education at CWCs. In general, OTSS provided much more detailed information and indicators than ISS, the Likert scale ISS provided more nuance to the indicators collected, and the binary ISS provided more rapid data but without nuance. In general, the three analysis methods confirmed that the key component of facility-based ITN distribution that needs the most improvement is logistics management. The specifics differed which will be explained under “Key Differences”, and this discordance is where the global community, especially MOHs and national malaria programmes, which are the ones to lead and implement supervision, can help to align monitoring priorities and agree on indicators.

### Key similarities

Both supervision approaches required a combination of record review and onsite observation. Record review was time-consuming, especially when reviewing the recording and reporting tools of large health facilities, such as hospitals. Onsite observations provided important insights into the dynamics between health workers and patients. All three supervisory approaches may have been affected by observation bias, which is detailed under the limitations section and which should be considered in analyses of supervision results by MOHs and national malaria programmes.

Additionally, both supervision approaches collected results on logistics data management and found that this component of facility-based ITN distribution was weak across facilities. From OTSS, the average logistics score was considerably lower than that for service data management (90% at ANC, 86% at CWC) and observations (94% at both ANC and CWC). Logistics data management performance was suboptimal across all collected data points (Fig. 4), and the highest result for any logistics-related question was 76% (Was the ITN inventory control card up to date, where the card was available?). From ISS, both types of data collection also showed low performance of logistics data management. For example, the Likert scale method found that only 55% of facilities received an outstanding score for “Inventory control card for LLIN is updated; matches physical count of stock today” (67% if including a performance score of “Good”) at ANC and 58% at CWC (72% if including a performance score of “Good”), and the binary scale approach found that only 52% of facilities had an inventory control card for ITNs matching the physical count of stock at ANC and 62% at CWC. By having clear standardized indicators on the assessment of logistics management, MOHs can more effectively allocate and plan the needed resources to improve facility infrastructure and stock management.

All three approaches also collected data on the health worker’s interactions with clients and consistently found results to be high. From OTSS, almost all observed eligible beneficiaries received an ITN, almost all observed issued ITNs were documented in the patient card and register, and the observed client was given education on malaria and ITN use and care (Fig. 5). From ISS, Likert scale data showed that 84% of facilities received an “Outstanding” score for issuing ITNs to 100% of ANC registrants (93% if including a performance score of “Good”) and 81% of facilities received an “Outstanding” score for issuing ITNs to 100% of eligible clients at CWC (94% if including a performance score of “Good”), and the binary scale data found 82% of facilities issued ITNs to 100% of eligible clients. By having clear standardized indicators on the assessment of health workers’ interactions with clients, MOHs can more effectively support district health teams on how to support their facilities and health providers in adherence to policies and guidelines.

Finally, any of these approaches and analyses could have benefited from using multiple data sources for decision-making. For example, one could have used DHIMS data to strategically allocate resources for ITN OTSS. If there had been a drop in ITN issuing rates in a specific region, one may have advocated to conduct ISS in that region to get a better understanding of the quality-of-service delivery and then follow up with OTSS if it’s found that quality of ITN services had indeed dropped or was performing lower than expected. In addition, supervision could have helped validate results from DHIMS. The analyses of observation indicators, for example, aligned with recent analyses of monthly DHIMS ITN services in Ghana that found over 90% of pregnant women at ANC received an ITN and over 90% of eligible children at CWC received an ITN. Accordingly, MOHs will find this sort of comparison useful as an additional check on performance indicators reported through national health information management systems.

### Key differences

The key difference between OTSS and ISS and, consequently, the indicators for monitoring quality of facility-based distribution, was in the details. OTSS provided very detailed information, while ISS (as an approach) provided more limited data which can limit decision-making. This difference was inherently based on the objectives of the two supervision approaches. OTSS was developed by malaria projects, particularly the PMI Improving Malaria Diagnostics (IMaD) project, expanded through the PMI MalariaCare project, and improved through the PMI Impact Malaria project. ISS, on the other hand, was developed by the GHS to combine supervision activities being conducted by the different health programmes (e.g., Maternal and Reproductive Health, HIV/TB, Malaria). However, MOHs could consider how a more detailed approach such as OTSS could have been used for follow-up of higher-level supervision approaches such as ISS to investigate underlying reasons as to why performance is low. In addition, the data generated from the different approaches provided insight into how facility-based services are doing, what areas must improve, and what is currently working well.

As a result, there were many more performance indicators that can be monitored through OTSS. These indicators were much more specific, and the data used to calculate these indicators could have been manipulated into varying performance indicators. For example, observation data could have been used to consider the percentage of eligible clients who received an ITN, the percentage of non-eligible clients who did not receive an ITN, and more generally, the percentage of health workers adhering to ITN issuing guidelines. This manipulation of data was not possible using ISS data. In addition, ISS lacked indicators measuring documentation of ITN issuing by the health worker, such as recording in the register, patient card, or tally sheet.

Supervision can provide additional information around data quality. However, among the two supervision approaches investigated in this paper, only OTSS assessed data quality. This difference could have been due to the fact that ISS assessed many components of health facility service delivery, not only malaria, and took longer to conduct than OTSS. Analyses of the OTSS data service and data management indicators insinuated that most reported data to the national DHIS2 were accurate (although there were some nuances that necessitate caution), which should be cross-referenced with other results from other data quality activities such as routine data quality assessments (RDQAs). These data were crucial to collect, as the OTSS data provided key insights that are missing from ISS. For example, service data management was generally high (Figs. 2 and 3), and 80–90% of facility-months had data with the register being equal to DHIMS. MOHs can consider how to integrate RDQAs with supportive supervision activities, which reduce the burden of visits and time spent at facilities and improve resource allocation and use.

Finally, another key difference was using a binary method versus Likert scale to measure performance. The ISS data that were collected in the format of a Likert scale provided interesting and nuances interpretations of the results. One may argue that this sort of data collection is important, as there were a lot of nuances between facilities. For example, it may not have been appropriate to compare the service delivery of a teaching hospital against a small clinic owned by a midwife. In addition, the Likert scale allowed for the consolidation of responses into groups (e.g., combining “Good” and “Outstanding” as a pass mark and “Poor” and “Average” as a fail mark), which may have yielded more favourable results. On the other hand, binary results as collected in both OTSS and binary ISS made data simpler to interpret and analyses simpler. However, the binary method results lacked nuance (e.g., education being marked as Yes/No compared to being done well, somewhat well, somewhat not well, and poorly done).

### Comparison of indicators

OTSS indicators provided more granularity into how the channels are performing. The indicators included scores, which can help decision-makers zero in on which areas of the facility need most support (i.e., should they focus on data management, logistics, and adherence to ITN issuing guidelines). The indicators also included specifics of each area, which enabled supervisors to provide tailored support to the health worker and the facility based on their strengths and gaps. For example, knowing whether the issue within logistics was related to the availability of an inventory control card or related to the inventory control card being kept up-to-date yielded different actions by the supervisor (e.g., supporting the facility to get new inventory control cards versus conducting an on-the-spot training on how to fill out the inventory control card). ISS indicators, however, provided higher-level insights into performance. This eagle-eye view helped GHS as a whole since fewer data to collect enabled a more integrated data collection approach (not only focusing on malaria, which also saved on resources) and provided the malaria programme some insights into which facilities might need more granular follow-up with fewer data collected, but it reduced the ability of supervisors to provide ITN-specific guidance and support.

### Feasibility

One key consideration to measuring the quality of facility-based ITN distribution was whether it can fit into existing quality improvement activities or whether it should occur on its own. The approaches in Ghana all showed that it is highly feasible to add ITN distribution indicators into supervision at health facilities. Particularly, since facility-based ITN distribution in Ghana occurs at ANC and CWC (as is done in many other countries), ITN-related questions can be added into existing ANC and CWC checklists. Similarly, questions related to assessing the data quality of ITN indicators can be added into existing data quality assessment (DQA) tools. In addition, the ITN-related questions in both the OTSS and ISS checklists were few in number, which reduces the added effort by supervisors to collect that data. Specifically for observation, the supervisors will already be witnessing ITN issuing (or lack of) as they observe the service when conducting supportive supervision of ANC or CWC.

## Limitations

There are limitations to be considered when interpreting the results in this paper. In addition, supervision activities also inherently have limitations that should be considered when making decisions using data generated from supervision of health facilities.

First, the service data management and logistics data management results generated through OTSS should be taken with caution. Within the EDS checklist, supervisors ticked whether the variance between registers and DHIMS was within ± 5% and whether the balance on the inventory control card tallied with the physical quantity. However, the supervisor also entered the numeric amounts per element. Automated calculations in the EDS DHIS2 and Excel did not always align with what supervisors reported (e.g., comparing whether the Yes/No mark reported by supervisors matched with an automated Excel calculation of the variance).

Second, although the digital checklists for OTSS and both versions of ISS included some skip logic to improve quality of the supervision data generated, they did not have any mandatory questions. Consequently, supervisors may have left some questions empty intentionally. This issue resulted in a lack of clarity of whether the question was intentionally left blank (e.g., not applicable, no data from register to report) or whether it was an accidental human data entry error. Though analyses were not done to calculate the percentage of questions left empty, it was observed that a notable amount was skipped, which may have affected the overall scores within the OTSS analyses and explained why the number of facilities assessed per questions differed within the ISS analyses.

Third, as noted in the second limitation, the digital tools used built-in skip logic to show and hide questions based on whether they are applicable using answers to previous questions. However, there were issues in the skip logic for the observation sections of the checklist in the initial round of OTSS that used the ITN module. This was fixed for the subsequent round. In addition, the digitized ISS checklists had such complex sections that, given the current wordings and layout, it was not possible to use skip logic for some questions that may not always be applicable.

Fourth, analyses have not been done to investigate whether the existing data is sufficient to draw conclusions about the results being representative of the national, regional, or district levels. It is possible that some of these results were representative, given how the Government of Ghana has scaled these supervision approaches nationally. At the same time, the proportion of assessments in each region differed greatly from the actual number of facilities in each region. In addition, although all regions are typically targeted for every supervision round (despite approach), some regions were skipped due to limited resources or timing. In addition, even if all regions could have been visited, it was more likely that not all districts (and even more so at the sub-district level) would have been visited given the large number of districts (and sub-districts).

Fifth, there is an inherent bias in observation questions. Health workers may behave better when a staff member from the government (e.g., NMEP M&E Specialist, District Health Officer) is present during an ANC visit by a pregnant woman. Health workers may not follow national policy and the expected steps during service delivery due to several factors, including shortening time with a client due to high client load and/or a staff shortage [25–27]. Consequently, this bias limits the usefulness of comparing the ITN issuing rates from observation with those from DHIMS.

Sixth, although some qualitative data were collected as part of these supportive supervision checklists and qualitative action plans are co-developed between facilities and supervisors, these data were not analysed beyond use at subsequent visits for following up on agreed upon action items by supervisors. Analyses of these qualitative data may yield patterns to the types of problems supervisors see at facilities or how common challenges are resolved. However, this analysis was not part of the scope of this activity.

## Conclusion

Distribution of ITNs at health facilities to pregnant women during ANC services and children under five years of age during visits to EPI clinics known as CWCs in Ghana serve as critical channels for getting ITNs to the most vulnerable populations. Multiple studies have assessed the quality of health facility services in sub-Saharan Africa by reviewing data quality and facility readiness, and supportive supervision has been presented as an effective method for improving malaria case management in sub-Saharan Africa. However, there is a gap in evidence about how supportive supervision can impact the quality of ITN distribution through health facilities and how to assess the quality of ITN distribution. Ghana’s multiple supervision approaches provided an opportunity to help fill this gap.

This paper provided an overview of multiple approaches and indicators used to measure quality of ITN distribution at health facilities in Ghana. The two supportive supervision approaches were described to provide context about how the data are generated. One approach collected data in two different ways (Likert and binary), so indicators and analyses were shown in three separate ways. The pros and cons of the approaches and how the data were collected were discussed. These supervision approaches provided data about how to measure and monitor the quality of facility-based ITN distribution, and at times they provided similar data. For example, all provided insights into correct adherence to issuing ITNs to eligible clients, the quality of logistics management, and education of clients or caregivers during the visit. There were also key differences. Only OTSS provided additional data on data quality, and ISS only assessed education at CWC. OTSS provided very detailed information, while the Likert Scale ISS provided nuance by capturing information beyond a binary which reflected a more nuanced understanding of performance than just Yes/No.

These analyses highlighted some critical recommendations and limitations. Digitization provided an opportunity to have robust data for which stronger claims about the current quality of ITN distribution at health facilities can be made, as supervision data already brings challenges, such as observation bias and being representative at national or regional levels. Nonetheless, the insights generated from supervision are important and bring much value to decision-making. Of note, record reviews (e.g., service data management and logistics data management) can provide accurate and meaningful information, supervision visits are an opportunity to get updated guidelines and documents to facilities, and supervision visits are also an opportunity to integrate with routine data quality assessments to improve optimization of resources.

The authors hope these analyses move the global community, especially MOHs and national malaria programs, to publish other analyses on the quality of continuous distribution of ITNs, especially through health facilities, and to agree on standardized performance indicators and analysis methods. Clearer understanding of what’s working and not working in continuous distribution of ITNs at health facilities will enable facility supervisors to more effectively support their facilities in providing higher quality healthcare services, particularly in ensuring all pregnant women at ANC and children at EPI receive an ITN. Additional publications from different countries will help fill in gaps in the literature about barriers to quality distribution of continuous distribution of ITNs including provider behavior, data quality, and logistics data management. The publication of additional analyses will allow cross-country analyses and systematic reviews which, in turn, will provide opportunities to provide recommendations with robust evidence and statistical power so that malaria programs can optimize these channels for routine distribution of ITNs.

## Data Availability

Datasets can be shared upon request due to privacy results of the facility-level data.

## Abbreviations

ANC: Antenatal Care
CWC: Child Welfare Clinics
DHIS2: District Health Information Software 2
DHIMS: District Health Information Management System
DHS: Demographic Health Survey
EDS: Electronic Data System
EPI: Enhanced Program of Immunization
GHS: Ghana Health Service
HNQIS: Health Network Quality Improvement System
IMaD: Improving Malaria Diagnostics
IPTp: Intermittent Preventative Treatment in Pregnancy
ITN: Insecticide Treated Net
ISS: Integrated Supportive Supervision
LLIN: Long Lasting Insecticide-treated Nets
LMD: Last Mile Distribution
MOH: Ministry of Health
MR2: Measles-Rubella Vaccine 2
NMEP: National Malaria Elimination Program
M&E: Monitoring and Evaluation
OTSS: Outreach, Training, and Supportive Supervision
PMI: President’s Malaria Initiative
RDT: Rapid Diagnostic Test
RDQAs: Routine Data Quality Assessments
WHO: World Health Organization
3 T: Test, Treat, and Track Policy
